# Brain organoids for addressing COVID-19 challenge

**DOI:** 10.3389/fnins.2022.1055601

**Published:** 2022-11-29

**Authors:** Jin Yu, Kailun Wang, Dalin Zheng

**Affiliations:** ^1^Department of Hematology, Panzhihua Central Hospital, Panzhihua, Sichuan, China; ^2^Department of Emergency, Panzhihua Central Hospital, Panzhihua, Sichuan, China

**Keywords:** brain organoids, COVID-19, organoids-on-a-chips, translational medicine, 3D culture

## Abstract

COVID-19 is a systemic disease involving multiple organs, and clinically, patients have symptoms of neurological damage to varying degrees. However, we do not have a clear understanding of the relationship between neurological manifestations and viral infection due to the limitations of current *in vitro* study models. Brain organoids, formed by the differentiation of stem cells under 3D culture conditions, can mimic the structure of tiny cell clusters with neurodevelopmental features in different patients. The paper reviewed the history of brain organoids development, the study of the mechanism of viral infection, the inflammatory response associated with neurological damage, the detection of antiviral drugs, and combined microarray technology to affirm the status of the brain organoid models in the study of COVID-19. In addition, our study continuously improved the model in combination with emerging technologies, to lay a theoretical foundation for clinical application and academic research.

## Introduction

The pandemic of COVID-19 has resulted in 635,538,583 confirmed cases and 6,594,050 deaths worldwide as of 31 October 2022, according to the World Health Organization.^1^ Almost 30–40% of patients have predominantly neurological symptoms such as headaches, strokes, and hyposmia (Gasmi et al., 2021). The construction of preclinical models to obtain first-hand information on possible mechanisms of viral infection between COVID-19 and neurological manifestations is of great urgency.

Common models for pathogen research include common two-dimensional (2D) cell and animal models. The models for COVID-19 include A549, Calu-3, Human neural progenitor cells-based 2D cells and mouse-based rodents, and even non-human primates such as rhesus monkeys, which may be efficient in studying possible mechanisms of viral infection and treatment (Blanco et al., 2020; Zhou et al., 2020). The 2D unit, which is the main model for current studies, also has some non-negligible problems. (1) Vulnerability to disturbances in the signal dynamics in the petri dish, which is detrimental to nerve cell growth. (2) Human nerve cells can show characteristic changes such as myelin formation and electrophysiology, while 2D cells lack these properties (Dehaene and Spelke, 2015). (3) 2D cultures emphasize individual cells, whereas the advanced functions of the brain are achieved through synaptic connections, and the regulatory functions of various glial cells cannot be presented in 2D cultures (Silbereis et al., 2016); Animal models are more widely used in the study of neurological disorders and altered consciousness, but again, there are some problems. (1) Not only has the human cerebral cortex evolved many more neurons than other mammals, but it has also formed functional structures such as the sulcus gyrus. More importantly, it has thinking activities not found in lower species (Herculano, 2009); (2) some medications may cause severe neurological damage and even death in patients due to drug accumulation during diseases treatment, but the aforementioned phenomena were not observed in preclinical animal models (Giandomenico and Lancaster, 2017). Given the above limitations of the models, we had to look for new *in vitro* research models, and therefore, we focused on organoids.

An organoid is an organ-like structure formed by the aggregation of stem cells in three-dimensional (three-dimensional, 3D) culture, containing cell types specific to the source organ that can reproduce a variety of functions such as secretion, filtration and even partial neural activity, according to the source organ function. Therefore, it displays the physiological functional structure of real organs *in vitro*, which then has great potential for replacing cell and animal testing (Sato et al., 2009; Lancaster et al., 2013; Lancaster and Knoblich, 2014). In the surge of COVID-19, organoids are contributing to host-pathogen interactions, to the study of differences in viral tropism of individual cells, and the kinetics of viral replication, especially in neurological infections, as organovir states ‘‘we want to bring organoids for virology to the next level.^2^ “Therefore, brain organoids play an essential role in the molecular pathology of virus-invading cells and the development of therapeutic drugs, and related research is in full swing (Table 1).

**TABLE 1:** Applications of brain organoids in virus.

Brain regions	Cell types	Patterning factors	Age of organoids when assayed	Virus type used	Infection details	References
Dorsal/ventral forebrain	Hipsc/ESC	Y27632, CHIR99021, SB431542, GM-CSF, A-83-01,LDN193189,dorsomorphin,WNT3A, IWR1e,XAV-939,SHH,SAG,NT3	35 days	ZIKV-MR776	Infected microglia-containing organoids are indicated by the significant morphological abnormalities and cell detachment.	Xu et al., 2021
Cerebral cortex(early-stage)	Human H9 ES (WA09)	bFGF,Noggin,BMP-4,VEGF, hLIF, Y27632, CHIR99021, SB431542, GM-CSF, Cyc A/SNH,BDNF,GDNF.	10 days	ZIKV-H/PF/2013	Organoids incubated with a virus inoculum are exhibited in time-dependent growth attenuation and significantly smaller	Krenn et al., 2021
Cerebral cortex	Hipsc	bFGF,Noggin,BMP-4,VEGF, hLIF, Y27632, CHIR99021, SB431542, GM-CSF, Cyc A/SNH,BDNF,GDNF.	14 days	ZIKV-H/PF/2013	Brain organoids are inoculated with ZIKV to induce a prominent change in organoid structure.	Pettke et al., 2020
Cerebral cortex	Hips	human recombinant FGF and EGF,	14 days	HIV-1 strain NLYU2	Both infected and uninfected microglia continued to infltrate into the hBORGs	Dos Reis et al., 2020
Cerebral cortex	Hipsc	bFGF, Y27632	15 days	HSV-1 strain F	Cerebral organoids are infected with different titer of HSV-1 in the culture medium for three day.	Qiao et al., 2020
Neocortical/forebrain	hiPSC lines (73 -56010 -01 SA and 73 –56010 -02)	bFGF, Y27632, CHIR99021, SB431542, LDN193189	12 weeks	HSV -1 strain KOS	Brain organoids are infected for 2 h, and then the inoculum is removed.	Abrahamson et al., 2020
Neocortical/forebrain	iPSCs	FGF,EGF, Y27632, CHIR99021, SB431542, LDN193189	12 weeks	HCMV strain TB40/E	The inocula of cerebral organoids are removed after 2h and then analyzed at days 3 and 5 post infection	Sison et al., 2019
ventricular/subventricular zone	AG14048 iPSCs	bFGF,Noggin,BMP-4,VEGF, hLIF, Y27632, CHIR99021, SB431542, GM-CSF, Cyc A/SNH,BDNF,GDNF.	>9 weeks	HCMV strains Towne	Brain organoids are exposed to virus for 24 h, and then medium is replaced.	Brown et al., 2019
Forebrain	HNSC.100	bFGF,Noggin,BMP-4,VEGF, hLIF, Y27632, CHIR99021, SB431542, GM-CSF, Cyc A/SNH,BDNF,GDNF.	>6 weeks	DENV-1 (Hawaiian strain)	Direct injection of the virus strain	Yoon et al., 2017

ZIKA, zika virus; HIV, human immunodeficiency virus; HSV, herpes simplex virus; HCMV, human cytomegalovirus; HIPSC, human induced pluripotent stem cells.

As a disease involving multiple systems throughout the body, a single brain organoid only statically reflects the pathological changes following direct viral action on specific cells. Moreover, since the human body is a dynamic and balanced system integrating multiple organs and a physiological microenvironment, how to combine the organ-like model of viral infection with existing equipment to realize the interaction between each organ and the microenvironment to study COVID-19 remains a concern. Therefore, we introduced the organoid-on-chips technology, which is a micro-physiological system built on a chip with microfluidics as its core, through the cross-fertilization of physics, chemistry, biology, materials science, engineering, micro-electro-mechanics and medicine, and other multidisciplinary science and technology fields (Reardon, 2015; Park et al., 2019). Organoid-on-chips technology is included in organ-on-chips technology, which replaces 2D cells with organoids and uses microfluidics to construct a unified whole *in vitro* to represent multiple organoids, biofluids, mechanical signals, and functional tissue interfaces (Wang et al., 2022). The use of this model allows for a more comprehensive understanding of the association between viruses and neurological manifestations based on existing findings, providing a new platform for discovering potential targets and developing effective treatments.

## The chronicles of brain organoids

Research on brain organoids dates back to 1907 when Harrison (1907) discovered that isolated sponge cells had the potential to spontaneously re-form organoids. Over the next century, many teams gradually progressed through 2D to 3D culture of embryoid and potential stem cells (potential stem cells, PSC) from experimental animals until a ROCK inhibitor sustaining human embryonic stem cells (human embryonic stem cells, hESC) passaging allowed hESC to survive through multiple passages (Martin, 1981; Thomson et al., 1998; Watanabe et al., 2005, 2007). Yoshiki, by adjusting the initial conditions of patterning factors and then the tissue will order folding and assembly spontaneously, generated a mammalian brain without external instructions in the Petri dish, unveiling the possibility for a variety of other systems to be studied while demonstrating the organoid as an *in vitro* model (Eiraku et al., 2008). Since then, many teams have continued to improve on culture protocol construction, culture identification, drug efficacy and toxicology testing, making organoids a hot spot in basic life science research, clinical disease simulation and new drug development (Eiraku et al., 2011; Lancaster et al., 2013; Sakaguchi et al., 2015; Watanabe et al., 2017; Mansour et al., 2018; Tao et al., 2018; Pellegrini et al., 2020; Popova et al., 2021).

## The pathogenesis of COVID-19 in brain organoids

COVID-19 is an infection induced by the synaptic protein on the envelope of the severe acute respiratory syndrome virus 2 (The severe acute respiratory syndrome virus 2, SARS-CoV-2), which enters the host cells along with receptors/co-receptors on the surface of the host cell membrane and other cofactors. Angiotensin-Converting Enzyme 2 (Angiotensin-Converting Enzyme 2, ACE2), transmembrane serine protease 2 (transmembrane serine protease 2, TMPRSS2), neomycin trichothecene 1 (neomycin trichothecene 1, NRP1) and furin/furin-like proteases are common receptor components, and the broad spectrum of ACE2 receptor hosts establishes its status as the primary receptor for neurological infections (Liu Z. R. et al., 2020).

### The mechanism of COVID-19 based on ACE2 receptor in brain organoids

When comparing the distribution of ACE2 in different *in vitro* models, the expression of ACE2 in neurons and astrocytes in brain organoid was significantly higher than that in 2d cultures, so a rational choice of *in vitro* models is a prerequisite for achieving the reliability of the study results. COVID-19 selectively infects astrocytes and radial glial progenitor cells in brain organoids, but there are also differences in infection rates between the two: nearly 50% in the former compared to 30% in the latter (Chen et al., 2020). In addition to the above-mentioned cells, it has been suggested that the virus is more plexiform cellophilic, but the detailed mechanism relies on Jacob et al. (2020) showing infection rates of up to 20% in choroid plexus-like organs with COVID-19 are accompanied by a large number of transcribed genes de-regulated. Genes related to cytokines and vascular remodeling were upregulated, whereas genes related to ion channels and intercellular junctions were downregulated. Similarly, Pellegrini and Lancaster (2021) further found that IL-6 produced by the virus through intrinsic immunity and C3 and C4 production by the complement system led to both studies. Both results suggested that ACE2-expressing choroid plexus epithelial cells are the main pathogenic mechanism of viral infection in brain organoids as the blood-brain barrier is disrupted by the recruitment of inflammatory factors (Sungnak et al., 2020).

However, ACE2 expression is lower in the neuronal cells, which make up the majority of brain cells, and the virus has a different proclivity for these cells (Qi et al., 2020). Some studies pointed out that the virus does not infect neurons, but several studies also concluded that the virus affects the upregulation of metabolism-related pathways such as cell division and the formation of a hypoxic environment at the foci of infection; or causes stress and direct damage to neurons by altering the distribution of Tau proteins in neurons and hyperphosphorylation (Song et al., 2020; McMahon et al., 2021); The above studies mentioned that viral infection interferes with metabolic processes, leading to changes in the microenvironment between normal and infected cells. The aforementioned studies suggest that viral infection interferes with metabolic processes, thereby affecting the survival of pericytes which constitute the neurovascular brain. Interestingly, viral mRNA may be 50-fold higher in pericyte-containing organoids, which act as a center of viral replication. In contrast, in controls without pericytes, no SARS-CoV-2 fragments are detected, which may be related to the fact that pericytes promote astrocyte maturation and mediate viral infection of cells (Ramani et al., 2020). Furthermore, it has been proposed that infection causes corresponding neurological symptoms such as headache and dizziness by rapidly decreasing the number of excitatory synapses of cortical neurons in brain organoids (Wang L. et al., 2021). In addition to the ACE2-mediated direct infection of neuronal cells described above, the nasal cavity, consisting of the sieve plate and olfactory epithelium, establishes a contact with the nerve center, and the severe acute respiratory syndrome virus infection of the nervous system via this pathway also provides new inspiration, that is, whether COVID-19 can also invade the nervous system along this pathway (Baig et al., 2020)? The expression level of ACE2, the key to viral invasion, correlates with the time of organoid culture. Bullen et al. (2021) notes that brain ACE2 expression was highest in organoids at 9 weeks, while McMahon et al. (2021) found that organoids cultured *in vitro* for 180 days had viral titers that could be diluted to 10^2^ PFU due to the wide distribution of ACE2 and that the amount of ACE2 was not constant. As the virus replicates in neuronal cells, ACE2 expression increases accordingly, which warns us that ACE2 mRNA levels do not represent ACE2 protein expression (Wang L. et al., 2021); it is worth mentioning that ACE2 expression levels also change if patients have comorbidities such as chronic diseases like hypertension and neurodegenerative diseases like Alzheimer’s disease (Cantuti et al., 2020).

### The mechanism of COVID-19 based on other receptor in brain organoids

Most of the aforementioned studies on nerve cell infection by COVID-19 have focused on ACE2, which is less expressed in olfactory cells. And the higher viral infection may be attributed to the high expression of the NRP1 co-receptor, which promotes viral interaction with a small number of ACE2 receptors to enhance viral entry into host cells when ACE2, TMPRSS2, and NRP1 are present together, but does not promote infection when only NRP1 is expressed. Meanwhile, the role of NRP1 depends on florin cleavage of the viral S protein to form a specific c-terminal sequence that promotes viral invasion of neuronal cells (Andrews et al., 2021). Similarly, in human cortical organoids, no significant ACE2 expression was observed in astrocytes, while in virus-infected cells, CD147 and DPP4 co-receptor expression were significantly higher in virus-infected cells, and the rate of viral infection also varied with the amount of co-receptor expression. Interestingly, co-receptors also induce an inflammatory gliosis-like injury (Wang et al., 2020). It has also been suggested that T lymphocytes with low levels of ACE2 expression are also susceptible to infection, which may be associated with CD147 receptor molecule-mediated endocytosis on the surface of T cells. Further studies are still needed to address whether it is another potential target for viral invasion (Blanco et al., 2020).

### The mechanism of inflammation of COVID-19 in brain organoids

There is also controversy regarding the neuroinflammation induced by viral infection via the aforementioned receptors (or targets). Some studies have pointed out that infection does not cause the production of neuroinflammation such as interferon (interferon, IFN; McMahon et al., 2021). But others have pointed out that low levels of IFN I and III with elevated chemokines and high expression of IL-6 are key to the development of neuroinflammatory symptoms, even more importantly, infected astrocytes are involved in neuroinflammation through upregulation of inflammation-related genes such as IFIT1, IFI44, and ISG15, and that genotoxic stress activation leads to nearly 20% apoptosis (Ramani et al., 2020). Inflammatory factors such as IL-1β, IL-6, chemokine ligand 5, and chemokine ligand 10 are significantly upregulated, compromising the integrity of the blood-brain barrier (blood-brain barrier, BBB). Local hypoxia produces an inflammatory response, which in turn lowers the threshold for tissue damage and thus exacerbates neurological damage, and these findings further expand our understanding of the pathogenic mechanisms of COVID-19 (Blanco et al., 2020).

## Screening therapeutic regimens of brain organoids in COVID-19

With an understanding of the pathogenesis of COVID-19, drug selection and vaccine development become particularly important in treatment and prevention. We all know that antiviral drugs generally take 8-12 years to develop, and even after hitting the market, they can be withdrawn at any time due to issues of efficiency, quality, targeting, and safety issues; Vaccines involve phase IV clinical trials and require constant adaptation to cope with the high variability of the virus, and as Feinberg says, ‘‘This is a world where we’re going to see infectious diseases we’ve never seen before, and we need to get really good at developing vaccines against them quickly.^3^ “And the time to market for vaccines is usually longer than for drugs, taking about 8–18 years. The urgent need to develop antiviral drugs and vaccines that use brain-based organs continues to be explained by the wide range of people involved in COVID-19, the high mortality rate, and the poor prognosis.

Given that ACE2 is a major molecule mediating infection, Monteil et al. (2020) attempted to inhibit viral infection by blocking the ACE2 receptor, and they used human recombinant soluble ACE2 (hrsACE2, human recombinant soluble ACE2) to treat virus-infected vascular and kidney organoids. They found that the rate of virus infection in vascular organoids was reduced by about 5-fold before and after the use of hrsACE2, while the effect was even more dramatic in renal organoids, even decreasing by nearly 1,000-fold. Since ACE2 is widely distributed in multiple tissues, including the brain, can hrsACE2 achieve the same anti-infective effect in infected brain organoids? In addition, in brain organoids with TMPRSS2 receptor expression, the serine protease inhibitor Camostat Mesilate, blocks viral S protein-mediated infection (Hoffmann et al., 2020). The EK1 peptide, an inhibitor of stinging proteins, inhibits endocytosis mediated by the binding of viral stinging proteins to cellular receptors at 40 μM, thereby inhibiting viral infection by interfering with the cell fusion process (Blanco et al., 2020). Not only are there drugs targeting each receptor molecule but the monoclonal antibody tocilizumab to ACE2 is also very effective and inhibits viral infection of the organoid even at 100-fold dilution (Song et al., 2020). Lv et al. (2021) found that using SARS-CoV-2 infected lung and small intestine organoids, including imatinib, mescaline, quinacrine hydrochloride and chloroquine, could inhibit virus invasion. Moreover, the effective concentration of mycophenolic acid in the four drugs could even be as low as 0.15 μM, and the animal model also verified the experimental results, enlightening us whether SARS-CoV-2 can first infect the lung, then there was a relay station for virus replication and transmission, and eventually led to brain infection through blood circulation remains to be further investigated. Sofosbuvir, an Food and Drug Administration-approved anti-hepatitis C drug, was used to prevent infection-induced neurotoxic changes because of its high homology to SARS-CoV-2 in terms of base arrangement. Mesci et al. (2020) chose 20 μm of Sofosbuvir to treat brain organoids infected with the virus and found that the drug significantly reduced virus replication and nerve cell death in brain organoids.

## The applications of COVID-19 in brain organoid-on-a-chip

Brain organoids reproduce, to some extent, some of the structures and functions of the human nervous system, but the lack of vascular endothelial cells and immune cells exposes us to the deficiencies of this model in simulating the host’s inability to initiate intrinsic and adaptive immune response after the pathogenic attack; simultaneously, the formation of neuronal networks in the brain is dependent on the regulation of physicochemical factors, and most existing protocols include chemical signals such as neurotrophic factors and hormones sequentially to induce nerve cell differentiation, migration and maturation, and eventually brain organogenesis (Yu et al., 2022). Without including physical signals in the culture. This may result in problems such as difficulties in the simulation and control of organoid tissues whose structure and function are not equivalent to the source tissue’s physical signals, including mechanical, electromagnetic, and optical signals. Additionally, because of challenges with modeling and control, organoids are often only maintained in static culture systems, even though both the growth of nerve cells and the production of myelin depend on the presence of physical signals (Ryan et al., 2021). However, more importantly, fluids are crucial in order to meet the high metabolic demands during organoid culture and to avoid inefficient ways of oxygen uptake and nutrient supply in static culture systems, such as passive diffusion of nutrient supply only. In a study of COVID-19 infection of the intestine, the expression of ACE2 increased significantly with the combination of physical signals such as fluid shear and mechanical peristalsis, laying the model basis for further studies (Bein et al., 2021).

The organic combination of developmental biology, bioengineering, and 3D stem cell culture technology to form a micro-processed cell culture device, the organ chip, depends on how to introduce and precisely regulate the physicochemical factors at the right time during each stage of model construction. Therefore, the existing organoid models can have a high match with the source tissues. It simplifies the major anatomical structures of the target organ and takes the corresponding cells for co-culture with novel materials and a controlled microfluidic environment, forming a model with high fidelity. The model is widely used, especially in alveolar epithelial cells, endothelial cells, fluid, air-fluid interface, and air sac structures on both sides that deform with respiratory movements together constitute the lung organ- on-a-chip, and then infection with the COVID-19 can better simulate the virus transmission process along the respiratory tract (Thacker et al., 2021). Since organoids composed of multiple cells produce coordinated mechanical activity in terms of traction and fluid shear that is significantly different from that of organoids formed by individual cells on chip. Combining organoids containing multiple differentiated cells with microfluidic systems to form organoids on a chip allows the construction of *in vitro* models with more significant advantages (Guo et al., 2021; Zhang et al., 2021).

**FIGURE 1:**
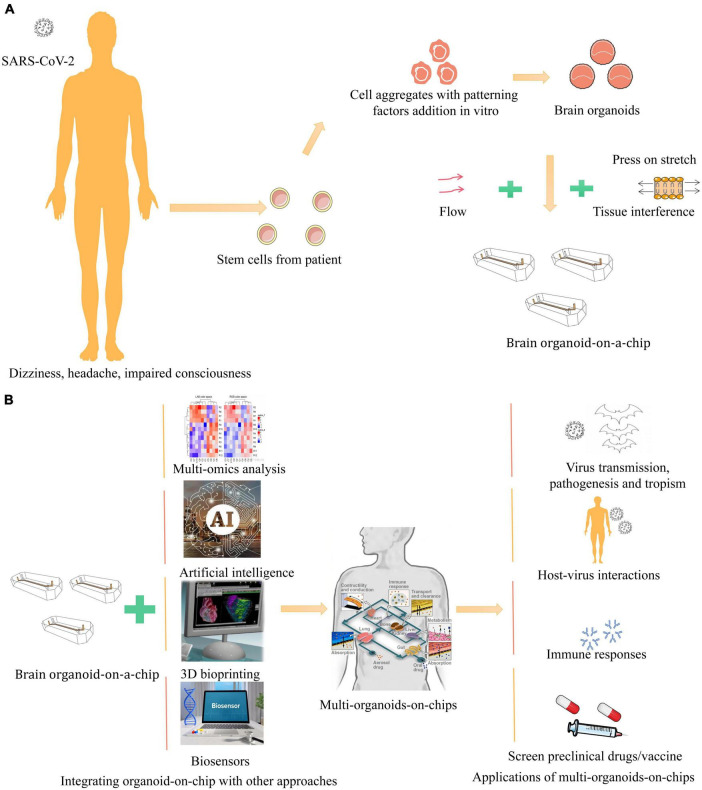
Schematic illustration of *in vitro* human organoid models for COVID-19 research. **(A)** COVID-19, clinically presents a wide spectrum of neurological complications, such as dizziness, headache, impaired consciousness (Blanco et al., 2020). Brain organoids are derived from human stem cells and aggregated with patterning factors addition *in vitro* (Chen et al., 2021). With fluid flow, stretch, and tissue interface to mimic cellular microenvironment on a bioengineered microfluidic cell culture device, recapitulating the brain organoid-on-a-chip (Park et al., 2019). **(B)** Integrating brain organoid and other organoids with other approaches like multi-omics, biosensors, 3D bioprinting, and artificial intelligence to generate multi-organoid-on-chips to provide new pathway for virus, which physiologically relevant model systems can be applied to study SARS-CoV-2 virus transmission, pathogenesis and tropism, host-virus interactions, immune responses, and screen preclinical drugs and vaccine (Sang et al., 2021; Wang et al., 2022).

For the current status of the COVID-19 pandemic, organoid microarrays have become the first choice in studying the potential pathogenesis of the virus. The lung respiratory membrane and blood-brain barrier communicate through the microfluidic chip. Interestingly, unlike the virus that directly infects brain organoids to destroy the integrity of the BBB, in this experiment, the virus first infects alveolar organoids on the chip and activates immune cells in the lung to trigger systemic inflammation, leading to brain endothelial damage and BBB dysfunction, validating the theory that COVID-19 is a multisystem infection. At the same time, this study mimics pathological changes in the viral host after infection at the organ level, which is difficult to achieve in other *in vitro* models (Wang P. et al., 2021). Moreover, even CD4 + T cells in the intestine of necrotizing enterocolitis (NEC, necrotizing enterocolitis) mice induce NEC-associated brain injury by releasing IFN-γ in brain organoids via blood circulation (Zhou et al., 2021). All these studies showed the obvious limitations of single brain organoids in studying systemic diseases, highlighting the need for multi-class organ microfluidic chips as an experimental research vehicle (Figure 1).

## Challenges and future prospect

Traditional *in vitro* models such as 2D cells and experimental animals have been more successful in the study of pathogens. However, for infections where 2D cells cannot mimic host-pathogen interactions and the host spectrum is limited to humans and cannot be performed in animal models, organoids have leaped to be a better choice under their homology with the source host and their high genetic and phenotypic match. Current studies on COVID-19 infected organoids have focused on the lung, especially when screening drugs using this model, not only have clinical therapeutic doses of certain drugs failed to achieve inhibition of viral infection in lung organoids but even shown experimentally from experiments in 2D cellular and animal models (Si et al., 2021). Although some patients with clinically significant neurological symptoms of COVID-19, most of whom patients exhibit neuroinflammation caused by a storm of inflammatory factors. Building a research system targeting brain organoids to demonstrate infection-induced immune responses and interactions between multiple organs to address these questions. The formation of a brain-organoid-on-a-chip, an automated platform by co-culture with mesenchymal and immune cells and other advanced approaches, in addition to fluidly connecting different organs into a whole, inspired us to build a “multi organoids-on-chips” to achieve the goal of immune response and the study of COVID-19 from a multi-organoids level (Skardal et al., 2017). In February 2022, the U.S. FDA formally approved the cold agglutinin syndrome drug Enjaymo for autoimmune demyelinating diseases based on preclinical efficacy data from human organoids, demonstrating that we can achieve the goal of ‘‘old drugs, new uses’’ by relying only on organoid models and providing clinical efficacy evidence for new drug indications.^4^ In addition to studying infection mechanisms and treatment options, the brain organoids on a chip can also be used to study the long-term effects of environmental factors such as nicotine that predispose to neurological disorders (Wang et al., 2018). And the use of organoid microarrays in vaccine research includes a significant increase in B-cell differentiation and specific IgG antibody production in tonsil organoids infected with live attenuated influenza vaccine and triple vaccine against measles, mumps, and rubella (Wagar et al., 2021); In contrast, lymphoid follicle organoids formed by B and T lymphocytes in human blood are similar to tonsil organoids in terms of cytokine secretion and immunoglobulin production after influenza vaccination. And with the addition of antigen-presenting cells, they can also be used to test immune responses induced by vaccines and adjuvants (Goyal et al., 2021). To research viral and vaccine effectiveness in real-time and combat the formation of mutant virus strains, brain organoid microarrays are therefore a valuable tool.

In terms of the current problems with brain organoids, the duration of brain organoids culture varies from weeks to months, with material selection, culture protocols, virus titers, and duration of infection effects varying between research teams (Yi et al., 2020; Zhu et al., 2022). Moreover, the affinity of viruses for brain organoids varies depending on the growth stage, and most organoids formed during culture are currently used only for studies related to the early stages of neurodevelopment, whereas the extended culture time allows brain organoids to have a broader scope of application, especially in the study of pathogenic infections, degenerative diseases. In addition, brain damage caused by mid-to late-stage chronic diseases, and the communication between nerve cells and the connections established at each synapse is also crucial for the reproduction of functions (Sidhaye and Knoblich, 2020). How to mimic viral recruitment of immune cells and initiate immune responses to clear viruses when combined with other pathogens to form a complex infection. And the use of specific hydrogels instead of animal-derived matrix gels can reduce the variability introduced by the compositions and batches (Liu H. T. et al., 2020). Since culture protocols vary from lab to lab and the culture period and size of organoids vary, it is also possible to separate organoids of a certain size by setting traps on microcolumn arrays to obtain uniformly sized organoids (Zhu et al., 2017). Currently, technologies such as multi-omics analysis, artificial intelligence, 3D bioprinting and biosensor provide new ways to efficiently study virus-induced diseases, and the combination of brain organoids with these approaches ultimately allows for study the virus transmission, host-virus interaction, immune responses, and the development of new therapies and vaccines (Yin et al., 2021). It is believed that with the improvement of culture technology and further research on viruses, organoid models can continuously provide new clinical ideas in the field of pathogen research, which is expected to solve the bottlenecks and troubles of clinical diseases and provide better application prospects for the development of academic research and translational medicine.

## Author contributions

JY and KW: conceptualization. KW and DZ: investigation. KW: resources. DZ: supervision. JY and DZ: writing—original draft. JY: writing—review and editing. All authors contributed to the article and approved the submitted version.

## Footnotes

1
https://worldometers.info/coronavirus

2
https://organovir.com

3
https://www.statnews.com

4
https://www.pharmacytimes.com
